# Dental neglect as a marker of broader neglect: a qualitative investigation of public health nurses’ assessments of oral health in preschool children

**DOI:** 10.1186/1471-2458-13-370

**Published:** 2013-04-19

**Authors:** Caroline Bradbury-Jones, Nicola Innes, Dafydd Evans, Fiona Ballantyne, Julie Taylor

**Affiliations:** 1 School of Nursing & Midwifery, University of Dundee, 11 Airlie Place, Dundee, Scotland, UK; 2 Preventive and Children’s Section, Unit of Dental and Oral Health, Dental School, University of Dundee, Park Place, Dundee, Scotland, UK; 3 NHS Fife, Greenfield Clinic, Willow Drive, Whyteman’s Brae, Kirkcaldy, Fife, Scotland, UK; 4 University of Edinburgh/NSPCC Child Protection Research Centre, Moray House, 3.15 St Leonard’s Land, Holyrood Road, Edinburgh, Scotland, UK

**Keywords:** Children, Dental, Neglect, Nurse, Oral, Public health, Qualitative, Threshold

## Abstract

**Background:**

Child neglect is a pernicious child protection issue with adverse consequences that extend to adulthood. Simultaneously, though it remains prevalent, childhood dental caries is a preventable disease. Public health nurses play a pivotal role in assessing oral health in children as part of general health surveillance. However, little is known about how they assess dental neglect or what their thresholds are for initiating targeted support or instigating child protection measures. Understanding these factors is important to allow improvements to be made in care pathways.

**Methods:**

We investigated public health nurses’ assessment of oral health in preschool children in relation to dental neglect and any associations they make with child neglect more broadly. A qualitative study was conducted in Scotland during 2011/12. Sixteen public health nurses were recruited purposively from one health region. Individual, semi-structured interviews were undertaken and data were analyzed inductively using a framework approach. Categories were subsequently mapped to the research questions.

**Results:**

Public health nurses assess oral health through proxy measures, opportunistic observation and through discussion with parents. Dental neglect is rarely an isolated issue that leads on its own to child protection referral. It tends to be other presenting issues that initiate a response. Threshold levels for targeted support were based on two broad indicators: social issues and concerns about child (and parental) dental health. Thresholds for child protection intervention were untreated dental caries or significant dental pain. Barriers to intervention are that dental neglect may be ‘unseen’ and ‘unspoken’. The study revealed a communication gap in the care pathway for children where a significant dental problem is identified.

**Conclusions:**

Public health nurses take their child protection role seriously, but rarely make a link between dental caries and child neglect. Clear guidance on oral health assessment is required for public health nurses. Establishing formal communication pathways between child dental care providers and public health nurses may help close gaps in care pathways. However, further research is required into how these communication mechanisms can be improved.

## Background

Child neglect is a significant issue in terms of prevalence and severity – it is the most common reason for a child to be made subject to a child protection plan in the UK - and there is indubitable evidence that it is harmful to children [1-4]. The National Society for the Prevention of Cruelty to Children (NSPCC) study on child maltreatment in the UK found that one in ten young adults had experienced serious neglect during their childhood [5]. Neglect is defined as the persistent failure to meet a child’s basic physical and/ or psychological needs, likely to result in the serious impairment of the child’s health or development [6]. A range of adverse health outcomes causally related to neglect is demonstrated significantly in both prospective and retrospective studies [7]. Disruption in attachment patterns and to neurobiological pathways means that neglected children carry a burden of long term consequences into adulthood; and potentially to subsequent generations. The economic burden to the UK caused by maltreatment is enormous, with particular long-term consequences for health and social services and the criminal justice system [8,9]. It is not just the UK though: in the United States the annual cost to the taxpayer from neglect is estimated to be more than $100 billion [10]. Child neglect also represents the majority of all maltreatment cases. In 2010/11 almost half (42%) of all registrations on Child Protection Registers in Scotland were for children suffering physical neglect [11] and similar trends are seen in the rest of the UK [12], Canada [13], the United States [14] and Australia [14]. Child neglect receives less attention than sexual or physical abuse (the neglect of neglect). Early intervention is crucial, but both recognition of, and responses to, neglect remain inconsistent.

Both the American Academy and the British Society of Paediatric Dentistry define dental neglect as being the willful or persistent failure to meet a child's basic oral health needs by not seeking or following through with necessary treatment to ensure a level of oral health that allows function and oral health (freedom from pain and infection) [15,16]. Dental neglect can result in the impairment of the child’s oral or general health or development [17]. Dental neglect may exist in isolation, however, there is increasing acceptance that untreated dental disease may be a useful indicator of broader child neglect [17,18]. Abused and neglected children have been found to have higher levels of tooth decay than the general population. In one Canadian case control study, five year olds with a history of maltreatment had experienced almost twice the number of caries lesions as children in the control group [19] and a similar US based investigation found the study group of abused/neglected five to 13 year-olds to be almost eight times as likely to have untreated, decayed permanent teeth than their controls [20].

Where there is widely available access to dentistry, such as free National Health Service (NHS) dentistry in the UK, but persistent failure of parents or carers to access dental treatment for their child’s tooth decay, this should be considered an alerting feature for practitioners to *consider* neglect [21]. Specific indicators include: repeated non-attendance for scheduled oral health assessments (dental checkups); attendance for emergency pain relief more than once; and requirement for dental extractions/care under general anesthetic more than once [17,22,23]. There are shared views from countries as far afield as the Czech Republic [24], Hong Kong [25], Philippines [26] and Australia [27], that childhood caries represents a significant public health issue. Perhaps unsurprisingly, this is especially the case in countries where national programs of oral health assessment and access to primary oral health care are absent [28].

Public health nursing was established in the UK in the mid-19^th^ century. Some countries have similar models of public health nursing to those in the UK, but in other countries the role does not exist. In the UK, all public health nurses are registered nurses with specialist qualifications in community health. Their role is an integral part of primary healthcare services, with a focus on prevention and health promotion. Every family with children under five has a public health nurse to offer support to families through the early years. The UK system of public health nursing assessment is based on a number of complex principles that involve ongoing assessment and prioritization [29]. Integral to this is the assessment and promotion of oral health among children and discussion of oral health with *all* parents and carers on at least four occasions during a child’s preschool years [30]. Targeted support and intervention is provided for families and children deemed to be in most need.

Because of their extensive contact with children, public health nurses’ role in child protection is widely recognized in terms of prevention; identification; intervention and support. However, it is known that there is variation in health professionals’ perceptions of thresholds of neglect [31]. It is also known that public health nurses use dental neglect as a proxy indicator of broader neglect in children [32]. Their role in the accurate, timely assessment of children for dental neglect means that they are potential catalysts in securing a child’s overall safety and well-being. Understanding how public health nurses assess oral health, particularly in relation to dental neglect, is thus an important part of the wider child protection agenda. However, what is not known is what public health nurses actually *do* to assess for dental neglect. This study sought to address this gap in knowledge.

### Aims & research questions

The aim was to investigate public health nurses’ role in assessing oral health in preschool children in relation to dental neglect. The research questions were:

1. How do public health nurses assess oral health?

2. What are the potential barriers to such assessment?

3. What threshold levels of dental decay are used by public health nurses as an indicator of the need for targeted public health nurse support?

4. What are the factors relating to dental neglect beyond which public health nurses initiate child protection intervention?

## Methods

Public health nurses’ role in identifying cases of child neglect as they relate to dental disease is an under-researched area. An exploratory research design was deemed to be appropriate because it aligns with the UK Medical Research Council’s (MRC) framework for complex interventions that emphasizes the need for exploratory investigations as a necessary precursor to future intervention studies [33]. In this qualitative study, we investigated the role of public health nurses in assessing oral health in preschool children in relation to dental neglect. Data were collected from a purposive sample of 16 public health nurses through semi-structured interviews. Data were analyzed using Ritchie and Spencer’s framework analysis approach [34].

### Ethics

Under new research governance procedures in the UK, NHS ethics approval was not necessary in order to conduct the study because it did not involve NHS service users or their relatives/carers [35]. However, the research protocol and study documentation were scrutinized by the Research and Development department of the relevant health board and permissions to undertake the study were obtained. Participants were invited to join the study and were given written information leaflets about it before informed and written consent was sought. This was gained from all participants.

### Recruitment

Public health nurses (n = 16) were recruited using purposive sampling. Purposive sampling is widely used in qualitative research as a means of recruiting participants who share experience of a certain phenomenon; in this case, the assessment of children’s oral health. A sample of 16 was deemed to be sufficiently large to allow for meaningful insights to be gained, yet manageable regarding the volume of qualitative data to be generated. To be included in the study, participants needed to be practicing within the field of public health nursing and working within the single designated health board in East Scotland.

### Data generation

Data were generated through semi-structured, 1:1 interviews undertaken between April and July 2012. To ensure consistency, interviews were conducted by FB. An interview schedule was used as a guide (Table 1) and as shown, participants were asked to recall an incident from practice where they had assessed the oral health of a child. All interviews were audio recorded and subsequently transcribed verbatim. To protect participants’ anonymity they were assigned a code from PHN1 to PHN16.

**Table 1:** Interview guide

**Primary prompt question**	**Follow-up questions**
1) Tell me about a situation where you assessed the oral health of a child	What did you do?
2) Now tell me about a situation where you were concerned about the oral health of a child	Why? Why were you concerned?
3) What type of assessments did you make of that child?	
4) What was the state of the child’s overall health?	What was the outcome?
5) What actions did you take?	
6) How did you feel about this situation?	

Data analysis was influenced by the framework approach of Ritchie and Spencer [34]. We chose this because it imposes structure on the analytical process while simultaneously allowing for the generation of inductively derived categories. Data were analysed using the sifting, charting and sorting of data that is characteristic of the framework approach. For consistency, FB analysed all the interviews; however analysis of each transcript was undertaken independently by another research team member. This was an important part of ensuring rigour. FB had been involved with data generation, so a whole-team approach to analysis provided a reflexive means of checking the ways that participant responses had been shaped by the interview process. The team then came together and emerging themes were discussed and revised until consensus was achieved. The agreed themes and sub-themes were subsequently mapped to the research questions as shown in Table 2.

**Table 2:** Data analysis chart

**Main theme**	**Sub-theme**	**Theme dimensions**	**# Cited**
**Question 1: How do public health nurses assess oral health?**			
**Observation**	Child’s teeth		15
		Opportunistic look in mouth	10
		Do not look directly in mouth routinely	9
		As part of holistic assessment	9
		Look in mouth if asked	9
		Look in mouth if concerned	7
		Observe decayed front teeth	6
	Family teeth		
		Parents teeth	11
		Siblings teeth	4
**Assessing parental attitudes**	Gauge parental interest in oral health		9
		If parental concern e.g. pain	7
		Parental dental phobia	6
	Parental feeding and weaning practices		
		Use of feeding bottles/dummies/juice/sweets	14
**Communication**	With parents		
		Ask if registered with dentist (routinely)	15
		Ask about teeth brushing	13
		Mention dental health services	10
		Use of assessment framework	7
		Ask about recent attendance with dentist	6
	Other communication channels		
		Knowledge of family/family history (e.g. through records)	7
		Through information from other professionals	4
**Question 2: What are the potential barriers to assessment?**			
	Public health nurse role		
		Not likely to be aware of problems further back in child’s mouth	7
		Public health nurses’ role is advisory	5
		Public health nurses not qualified to look in children’s mouths	4
		Not top of public health nurses’ agenda	4
	Parental expectation		
		Parents do not expect public health nurses to assess dental health	7
		Parents might consider it intrusive to look in child’s mouth	4
		Dental health - a very ‘sensitive’ subject	3
		Tension between choice and protection	3
**Question 3: What threshold levels of dental decay are used by public health nurses as an indicator of the need for targeted public health nurse support?**			
**Threshold levels for support**			
	Concerns about other social issues		
		Homelessness/drugs/domestic abuse	10
	Concerns about dental health		
		Concerns about child’s teeth	5
		Concerns about parent’s teeth	4
**Responses to identified need**			
	Issue dental packs		
		Additional issuing of dental pack	6
	Referral		
		Referral to dental services	10
**Question 4: What are the factors relating to dental neglect beyond which public health nurses initiate child protection intervention?**			
**Threshold levels for concern**			
	Concerns about other social issues		
		Dental decay alone is not a child protection issue	12
		Dental decay is a marker of broader neglect	11
	Concerns about dental health		
		Untreated dental caries or pain	10
		Not taking child to dentist	7
**Responses to concern**			
	Referral to dental services		
		Facilitate further dental appointment / attendance at appointment	7
		Repeated referrals to dentist	2
	Notifying another agency of concern /sharing information		
		Include or consider including information within child protection reports / risk assessments	8
		Notify (or consider notification) to social work of concern	2
**Barriers to intervention**			
	Communication and Feedback		
		Poor liaison between services	6

To supplement our qualitative analysis, we included a summation of how many public health nurses cited each main theme, sub-theme or theme dimension. For example, of the sixteen public health nurses who reported that they assess oral health through observation of a child’s teeth, ten stated that they do this opportunistically; nine told us that they look if asked; seven look if they have a concern, et cetera (Table 2). Of course, most public health nurses engage in multiple assessment practices which is reflected in the tally. In this qualitative study the figures are intended to provide an impression of the salience of a theme, rather than to make any statistical claims.

## Results

The study findings are reported with reference – and response - to the research questions.

### How public health nurses assess oral health in preschool children

Public health nurses in this study assessed oral health via three mechanisms: observation, parental attitude and communication. Observational assessments were primarily through direct noting of the condition of a child’s teeth. For most public health nurses in this study (n=10), this tended to be opportunistic, rather than a planned activity, for example:

I would just look at their teeth as I was chatting to the children. PHN 6

Just a smile… a smile of a child you can sometimes see things aren’t as they should be. PHN7

It’s the time during a home visit, you know, to go up to a child and say, ‘let me see your smile’ and doing it that way. PHN 15

For most participants (n=10), observation tended to be opportunistic, rather than a planned activity, for example when a child laughed or smiled. For many, direct observation was deemed to be beyond their sphere of practice:The majority of participants (n=11) reported that they also use parental dental health as a proxy indicator of the likely condition of a child’s teeth:

It’s not something I would do. PHN 8

I wouldn’t say it’s my role to look in a child’s mouth. PHN 9

No never, never because I wouldn’t know what I was looking for… PHN 12

[If mum] has got very decayed teeth herself I suppose that is another indication for me to be alarmed about what is going on with the children’s teeth by looking at the parents. PHN 3

I would always look at the parents’ dental health, because it is much more obvious at times. You know particularly looking to see what kind of state their teeth are in. That would be one of the first things that I would look at. PHN 4

Her own dental health and the care of her own teeth is going to reflect how she’s going to look after her child. Certainly my experience of the mother whose teeth are poor, are the ones I would look at in the child. PHN 6

Assessment of parental attitude was the second domain relating to how public health nurses assess children’s oral health. Feeding and weaning practises were cited by most participants (n=14) as key issues, particularly the use of bottles, dummies/pacifiers, juice and sweets/candies. Parents’ own experiences were also cited and six participants alluded to parental dental fear as an alerting risk factor for dental decay. Finally, regarding communication, most participants reported using discussion with parents on oral health issues to inform their assessments, such as asking about registration with a dentist (n=15) and while advising about teeth brushing (n=13):

Even if the child has no teeth I’ll say to her [mother] ‘still brush the gums and just get the child used to having the toothbrush in their mouth’. PHN5

Assessment of oral health issues was not confined to the family. A small number of participants (n=4) reported that they also discussed with other professionals and used information documented within family records to inform their assessments. Overall, findings pointed to a range of methods used by public health nurses in the study to inform their assessment of dental health. There were, however, some actual or potential barriers to assessment.

### Barriers to assessment

Barriers to assessment were concentrated around issues of public health nurses’ role and parental expectations around that role. Just under half the participants (n=7) said that they were unlikely as public health nurses to look into a child’s mouth and, therefore, would not be aware of any dental problems with a child’s back teeth unless the parent raised a concern. Other barriers were cited explicitly around parameters of the public health nurse role, with five participants stating that their role in dental health was advisory, for example:

I think the role is much more of an advisory one and offering advice about brushing and the effects of diet and carbonated drinks on children’s teeth and offer suitable alternatives. PHN 11

As indicated in the following excerpts, a child’s oral health status tends to be part of the ‘bigger picture’ of factors present for children and at the time of assessment, may not be top of the public health nurse’s ‘agenda’:

You would just see them ad-hoc at clinic and it [oral health] may not be on the top of my agenda. PHN 4

It’s probably not the top most of your mind when you are going into these families. You know, the basics there, are they safe, are they eating, are they growing, are their needs being met? So it’s not always your thought, ‘Oh by the way can I have a look in your mouth?’ PHN 14

Issues around parental expectations highlighted some interesting perspectives. Seven participants explained that they did not look in children’s mouths routinely, because this was not expected by parents. Moreover, findings indicate that some believed that to do so, may be construed by parents as being intrusive:

I think it would depend very much on the parent and it would depend on the parent’s attitude to services and authority and some parents I think would find it quite intrusive. PHN 1

It’s quite an intimate thing to look inside someone’s mouth. PHN12

I suppose there maybe is a little bit of a feeling of that as well, that it’s maybe being a little bit intrusive or a little bit invasive. PHN 15

The issue was also raised regarding dental health as a sensitive issue. This called for careful balancing regarding the need to make appropriate assessments of a child’s health and the need to foster positive relationships with parents:

It’s trying to do it a bit more subtly, because you want to see them again. You don’t want them to say, ‘I’m not going to see her again’ PHN 7

It’s really difficult because health visiting is a service that’s offered to everyone but no-one has to let me in [to their house], no-one has to uptake that service, they can say no. PHN 12

### Threshold levels of dental decay that indicate the need for targeted support

We found that, although levels of dental decay were not directly assessed by the public health nurses, they nevertheless considered it part of their decision-making regarding targeted support. The nurses used surrogate measures as proxies for dental health based on the two broad indicators of concerns about dental health and social issues. Ten participants cited social determinants such as homelessness, poor housing, domestic abuse and parental substance misuse as alerting issues:

We are looking at their development, parenting styles, emotional, social, play all that kind of thing and also physical well-being. So yes, it’s just part of the general assessment. PHN 5

The children with the more problematic dental health are the children where there’s other issues are going on… other issues of neglect or other issues for the mother, maybe the mother’s got mental health problems or other issues so environmental issues, social issues, other health issues. PHN 6

You’re looking at the risk factors… whether the parents are substance misusers, victims of domestic abuse, sexual abuse and just, their own family history. PHN 13

The public health nurses who took part in the study appeared to place emphasis on the broader, sociological influences on children’s health in assisting them in their assessments:Their interventions regarding targeted support consisted of two main strategies: provision of additional resources to promote dental health and referral to dental services.

The sort of things that would worry me particularly would be relationship issues, mental health, poor social circumstances in a damp house or overcrowding or a quick change of address. PHN 6

I think the obvious ones [concerns] are probably domestic violence, alcohol and drug misuse, probably single mothers who are unsupported… young mothers… PHN 7

Several times I would leave toothbrushes and toothpaste. PHN 3

I advised mum about the importance of going to the dentist and advised mum about the importance of getting her to brush her teeth and because we have access to dental packs I gave her one of the dental packs. PHN 16

### Factors beyond which child protection intervention is initiated

Findings show that untreated dental caries or significant dental pain are threshold levels for child protection intervention. In such cases, referral to dental services and sharing information with relevant partner agencies were the primary interventions employed by the public health nurses who took part in the study. Although the majority (n=11) reported that in their experience dental decay was most often a marker of broader neglect, a similar number (n=12) expressed the opinion that dental decay alone would not necessarily raise a child protection concern. There were two key indicators for when a child protection intervention may need to be considered: a child suffering from untreated dental caries or significant dental pain (n=10) and parents failing to take their child for dental care after being advised (n=7):

Well obviously if the child was in pain, if the child had any pain and the parent wasn’t attending to that pain. That would be child protection concern. PHN 6

[Child protection intervention may be needed] if there are no… if the family are not registered with a dentist… if they’re not accessing a dentist or there is evidence of poor oral health. PHN 15

In this study, public health nurse interventions in response to child protection concerns consisted of two main strategies: referral to dental services and sharing information with relevant partner agencies. Almost half of the public health nurses interviewed (n=7) indicated that they would facilitate either further dental appointments or the child’s attendance at appointments. Interestingly, whilst they recognized the issue, the referral was to dental services, but they did not mention concurrent referral to child protection services. Eight public health nurses reported that they would include (or consider including) information regarding the child’s dental health within child protection reports or risk assessments shared with partner agencies:

[I state it] very clearly in every report… it would be very clear that you have provided them with the information for a dentist and to date they’ve still not registered or they have registered but not gone. PHN 9

I think if it was part of an overall picture of neglect and you knew it was a major issue then you would have to [include it] when you were doing the report. PHN11

Regarding communication, many participants reported that they are reliant on parental reporting of attendance at dental services and outcomes for children, rather than through formal liaison channels with other agencies, hence:

I’ve never had a phone call from a dentist to say this family have come and I’m appalled or you know, I’ve never had a phone call from a dentist. PHN 9

I’ve made quite a few referrals [to dental services] and I’m just thinking, you know, ‘What’s happened? Have they been seen or have they not? Have they attended?’ PHN 14

## Discussion

Until now, the means by which public health nurses make assessments of children’s oral health in the UK has been largely un-investigated. This study has provided valuable insights into the processes involved. Findings show that public health nurses in the study rarely looked directly in a child’s mouth to assess dental health status. Public health nurses *did* make assessments, but rather than direct observation, they used a spectrum of proxy indicators, such as parental dental decay, poor dietary habits, and dental practices as well as parental attitudes towards oral health, as the basis for assessment.

Primarily, assessment was undertaken through opportunistic scrutiny of a child’s teeth, but we also found that the majority of public health nurses used parental dental health as a proxy indicator of the likely condition of a child’s teeth. Interestingly, a number of public health nurses in our study said they did not know what to look for in a child’s teeth, but nevertheless, they were prepared to base their assessments on what they could see of the parents’ teeth. Although this may appear a little strange, it is known that dental health behaviours in parents reflect the care that they then give to their children [36]. So it is likely that an assessment of poor parental dentition is a reasonable indirect marker for low tooth brushing and oral health habits carried out for young children by their parents.

It is interesting that the public health nurses in our study used front teeth as indicators of dental health. Early Childhood Caries (ECC) (previously called Nursing Bottle Caries) seems to be declining in prevalence but is still seen in children in Scotland [37]. The American Dental Association (http://www.ada.org/2057.aspx) define it as the presence of one or more decayed, missing or filled tooth surfaces in preschool-age children. It commonly presents as the front teeth becoming very decayed, often as a result of a sugary substance being placed in the child’s bottle. However, for the majority of children with dental caries it will be most frequently distributed towards the back of the mouth, in the molar teeth. In addition, most abscesses/ swellings and sinuses present around the molar teeth and because of the padded soft tissues of the cheeks around that area, are difficult to detect without a clinical examination involving looking inside the child’s mouth. Taken together, this means that public health nurses may have a very limited picture of a child’s dental health. An opportunistic look at the child’s front teeth as they are talking or smiling, reduces the chance of the presence of dental caries being detected until it causes cavitation of the front teeth. This is a late stage of presentation.

Observation however, constituted only part of the assessments made by the public health nurses. Most public health nurses questioned parents about current practices around oral hygiene habits and dietary practices. This action is entirely appropriate given that statistically significant correlations have been found between dental caries experience of children and their oral health-related habits [25]. As well as serving as an assessment of knowledge within the family, the public health nurses used this form of assessment as a way of gauging parental knowledge and attitudes towards oral health. Again, such practices are likely to be worthwhile. Parents’ own experiences and dental phobia can be an alerting risk factor for dental decay [3] and parental dental knowledge has a significant correlation with a child’s dental caries experience [25]. For this reason, increasing parents’ knowledge of proper feeding habits and oral health practices is deemed to be important [28].

Untreated dental disease has been noted to be a useful indicator of broader child neglect [17,18] and public health nurses have previously been found to use dental neglect as a proxy indicator of general neglect in children [32]. This is supported by the findings from our study where the public health nurses indicated that they perceived poor dental health in children to be a marker of broader neglect. For the public health nurses in this study, there were two key indicators that a child protection intervention may need to be considered: a child suffering from untreated dental caries or a child with significant dental pain where parents had failed to take them for dental care having been advised. This is in line with the markers recommended for acting upon in current guidance [21-23,38]. Whilst our findings support those of others and show that public health nurses would act in line with recommendations, there is a communication pathway break as they are not routinely made aware, by dental authorities, as to when a child has active dental disease. This has implications for public health nurses’ opportunity to engage in appropriate, timely follow-up.

The public health nurses in our study indicated that dental neglect is rarely an isolated issue that leads on its own to child protection referral. From this it may appear that there is disconnect between the acknowledgement of dental neglect as a marker of broader neglect and lack of initiation of child protection intervention. Dental neglect is considered to be part of a mosaic of issues associated with a neglected child and it tends to be other presenting issues that initiate a response in the first instance. There is perhaps confidence among public health nurses that neglect will be picked up through mechanisms other than through oral health assessment; and once recognized then it might be assumed that dental neglect could also be an issue. This reflects the apparent understandings of public health nurses, that dental neglect is part of a much broader picture of neglect. However, it appears that opportunity to look for decayed teeth in a young child and use this as a clinical marker for general neglect is currently not being maximised.

Consistent with other studies on neglect, concerns about neglected children were often perceived by public health nurses to be difficult to grapple with, as the chronic nature, coupled with the difficulty in demonstrating the potential harm of neglect, are significant and intangible [31,39,40]. Severe dental neglect constitutes actual harm and may be confirmatory of the more general concerns that tend to be intractable in neglect. There is more research to be undertaken to ascertain whether dental neglect is recognized by public health nurses prior to more general concerns about a child; and if it is, how they respond.

Findings from this study indicate that dental neglect can remain ‘unseen’ and ‘unspoken’. The former may be attributable in part to the oral health/disease indicators used by public health nurses being too blunt to detect dental neglect. It may also be because the mouth is rarely inspected directly making it not possible for insidious intra-oral disease to be detected. This group of public health nurses did not consider it appropriate to carry out a clinical examination of a child’s mouth and felt that they were not qualified to diagnose dental problems. Dental neglect may be ‘unspoken’ in the sense that public health nurses in our study expressed frustration and concern about communication deficits between professional groups, particularly regarding a child’s (non-) attendance at dental services. They felt this had an impact on their assessments of need, planning and follow-up of proportionate interventions. The unseen and unspoken aspects of our findings are important because when public health nurses become aware of dental neglect, they take a number of decisive actions to protect a child such as assessing for broader neglect, referral to dental services, and initiation of child protection intervention where appropriate. Public health nurse interventions in response to child protection concerns consisted of referral to dental services and sharing information with relevant partner agencies. The response to these issues – that they identified as child protection concerns - therefore largely consisted of making a referral to dental health services and *not* as a specific child protection response. This may be appropriate, but it conflicts with their separate reports of using untreated decay and failure to take a child for treatment when they are in pain, as triggers for child protection interventions. This has implications for education and awareness raising among public health nurses, in terms of dental neglect being considered as a marker for broader issues and potential child protection concerns.

Direct communication with dental services did not seem to be routinely used as a part of the assessment process when there was no particular concern about a dental problem involving pain or infection that needed to be managed. Powell and Appleton [41] emphasize the importance of public health nurses’ recognition of a child’s attendance at routine health appointments. They called for a re-conceptualisation of the phrase ‘*did not attend*’ to one of ‘*was not brought*’. What is clear from our study is that lack of any clear pathway for feedback on this does not allow public health nurses to know if a child ‘was not brought’ to dental health services, nor if they did attend and whether it was a serious case of dental neglect. This communication issue might be a common problem as oral health is often seen as separate to children’s general health [42]. Identification and instigation of local solutions would overcome this problem and could comprise, for example, salaried dental services, public health dentists and public health nurses working together to identify possible strategies.

The public health nurses alerted us to a post-referral gap in the care pathway and lack of feedback from dental care services regarding a child’s attendance or non-attendance. Typically they are not made aware of (missed) appointments and as a consequence are unable to plan further intervention, such as facilitating future attendance. One particular break in the pathway of care was identified for children where a significant dental problem was identified and the public health nurse initiated a dental referral. Although they could do so, there was no established pathway for the public health nurse to receive information back from dental care professionals as to whether the child had attended for treatment or not. As a result, a child whose medical/dental needs are *not* being met might never be picked up because the public health nurse was unaware that the child had not attended a dental appointment which was likely to be for management of infection and pain. It was quite possible that the public health nurse might not even have been made aware that the dental professional had arranged for general anesthetic for the child. This is a key finding and clear pathways are needed between public health nurses and dental health professionals.

Overall, findings show a range of assessments made by public health nurses and also some of the actual or potential barriers to assessment. Public health nurses made decisions about targeted support based on two broad indicators: social issues and concerns about dental health. Social determinants such as homelessness, poor housing, domestic abuse and parental substance misuse were alerting issues. Interventions regarding targeted support consist of two main strategies: provision of additional resources to promote dental health and referral to dental services.

### Limitations

The study has provided new insights into public health nurses’ assessments of oral health in children, specifically regarding dental neglect as a marker of broader neglect. However, there are several limitations that need to be acknowledged. Firstly, this was a qualitative study that drew on perspectives from a purposively selected sample from one region of Scotland. Although the sample size was quite large for a study of this scope, it was by no means representative. Therefore, like most other qualitative studies, transferability to other contexts needs to be considered thoughtfully.

Aligned with the above, a second limitation is that from our purposive sample of sixteen public health nurses, we have used summation to illustrate how many of them raised that particular issue. We could have used terminology associated with most reporting of qualitative studies, such as ‘many’, ‘several’, or ‘few’. However, this type of ‘verbal counting’ has been criticized for failing to provide meaning in the context of the research [43]. With this in mind, because the process of thematic charting of data allowed us to capture numerical frequency, we chose to use this in a meaningful way and state the actual numbers. We emphasize that from this, we do not seek to make any claims for generalizability.

Finally, practices, policies and public health nurse roles vary considerably across regions and countries. It may be that the experiences of assessment described by the public health nurses in our study are UK-centric - or even Scotland-centric - and thus fail to resonate with those from other locations. Again, this calls for caution regarding transferability.

### Implications for practice and research

An integral part of our research design was to conclude the study by sharing and testing findings with relevant stakeholders. On completion, therefore, we invited a range of colleagues from public health nursing, dental and voluntary services to attend a seminar. The purpose was to share the study findings and discuss and debate their implications for practice with those for whom they would have greatest clinical relevance. The stakeholder meeting was attended by 25 people and the implications for practice stated here are informed by the discussions at the event.

Regarding the public health nurse role, it may be appropriate for them to be able to follow up more rigorously whether children have had a dental examination, especially where dental neglect is suspected. However, clear guidance for public health nurses as to what to look for and how to look for it is required, with consistent follow-through. Related to this, training on oral health assessments may be useful and is something that the public health nurses at the stakeholder event viewed with enthusiasm. In particular, recognizing very early signs of dental decay may alert nurses to child neglect before it is recognized from other indicators. To test this hypothesis an intervention programme is required. An improved two way communication pathway between child dental care providers and public health nurses is important. In this, dental health services have a responsibility to communicate with public health nurses and provide feedback regarding a child’s attendance status.

Regarding research, further investigation is necessary to test a guidance tool for public health nurses in assessing dental neglect and in engaging in appropriate follow-through. Whilst dental neglect is seen as a proxy indicator for broader neglect, the uni-directionality of this in our public health nurse sample needs to be tested in a larger population. Research into how communication mechanisms can be improved regarding children’s dental health is also needed.

## Conclusions

This study has provided new insight into public health nurses’ role in the assessment of oral health in preschool children in relation to dental neglect. It has highlighted that dental neglect, whilst taken seriously by public health nurses, is not easily assessed or well defined in terms of thresholds. Public health nurses use three mechanisms to assess oral health: 1) a range of proxy measures; 2) opportunistic observation; 3) discussion with parents. Dental neglect may be ‘unseen’ (unidentified) and ‘unspoken’ (not communicated). The unseen and unspoken aspects of our findings are important because when public health nurses are aware of dental neglect they take a number of decisive actions to protect a child such as assessing for broader neglect, referral to dental services; and initiation of child protection intervention where appropriate. In the area of the UK in which the study was conducted, our findings have highlighted a gap in the care pathway for children where a significant dental problem is identified, particularly regarding interdisciplinary communication.

Dental neglect is rarely an isolated issue that leads on its own to child protection referral. It is considered to be part of a mosaic of issues associated with a neglected child and it tends to be other presenting issues that initiate a response in the first instance. This reflects the apparent understandings of the public health nurses who took part in our study, that dental neglect is part of a much broader picture of neglect. Although they take their child protection role seriously, a prospective link is rarely made between dental caries and child neglect. Clear guidance for public health nurses as to what to look for - and how - is required. Improved two-way communication between child dental care providers and public health nurses is necessary to close any gaps in care pathways. Despite the local nature of this study and the associated limitations, the findings and the issues that have been highlighted are translatable to other settings. This is irrespective of country or context.

In preparing this manuscript we have adhered to the RATS guidelines on qualitative research (http://www.biomedcentral.com/ifora/rats).

## Competing interests

The authors declare that there are no competing interests.

## Authors' contributions

DE, NI, CB-J, JT conceived of the study and participated in its design and coordination. FB undertook all data collection. All authors undertook data analysis. CB-J produced the first draft of the manuscript. FB, DE, NI, JT edited the draft manuscript. All authors read and approved the final manuscript.

## Authors' information

The study was undertaken as part of a programme of research being undertaken in Scotland as a collaborative venture between the University of Dundee (Schools of Dentistry and Nursing & Midwifery), NHS Fife; and the National Society for the Prevention of Cruelty to Children (NSPCC). CB-J has a clinical background as a public health nurse and is an experienced qualitative researcher. She has undertaken research involving vulnerable populations and has a specific interest in the issue of child abuse and neglect. NI has extensive research experience in paediatric dentistry, including studies involving children in primary care environments. She has played a key role in developing national guidelines in Scotland for childhood dental caries, which included guidance on managing children with dental neglect. DE is a Consultant in Paediatric Dentistry with extensive research expertise with children in the area of children’s dentistry – both for dental trauma and dental decay. He has chaired two national guideline development groups on management of dental caries in children. FB has six years’ experience of public health nursing and seven years previous experience as a School Nurse. She was a founder member of the School and Public Health Association (SAPHNA) in the UK. She currently holds the Child Protection Trainer role within NHS Fife, Scotland. Her clinical expertise in child protection adds a crucial practice dimension to this clinical – academic research collaboration. JT holds a position as the inaugural Chair in Child Protection at the University of Edinburgh/NSPCC Child Protection Research Centre. She is an internationally recognized expert in the area of child protection, with a wide range of publications in the fields of child and public health, child abuse and protection, social work and health services research.

## Pre-publication history

The pre-publication history for this paper can be accessed here:

http://www.biomedcentral.com/1471-2458/13/370/prepub
